# Structural and Functional Dynamics of *Staphylococcus aureus* Biofilms and Biofilm Matrix Proteins on Different Clinical Materials

**DOI:** 10.3390/microorganisms7120584

**Published:** 2019-11-20

**Authors:** Anna K. Hiltunen, Kirsi Savijoki, Tuula A. Nyman, Ilkka Miettinen, Petri Ihalainen, Jouko Peltonen, Adyary Fallarero

**Affiliations:** 1 Pharmaceutical Design and Discovery (PharmDD) Group, Pharmaceutical Biology, Division of Pharmaceutical Biosciences, Faculty of Pharmacy, University of Helsinki, Viikinkaari 5, 00014 Helsinki, Finland; anna.k.hiltunen@helsinki.fi (A.K.H.); kirsi.savijoki@helsinki.fi (K.S.); ilkka.miettinen@helsinki.fi (I.M.); 2 Department of Immunology, Institute of Clinical Medicine, University of Oslo and Rikshospitalet Oslo, 0372 Oslo, Norway; t.a.nyman@medisin.uio.no; 3 Laboratory of Physical Chemistry, Åbo Akademi University, Porthaninkatu 3-5, 20500 Turku, Finland; petri.ihalainen@metgen.com (P.I.); jouko.peltonen@abo.fi (J.P.)

**Keywords:** *Staphylococcus aureus*, biofilm matrix, clinical material, exopolysaccharide, proteins, surfaceome

## Abstract

Medical device-associated staphylococcal infections are a common and challenging problem. However, detailed knowledge of staphylococcal biofilm dynamics on clinically relevant surfaces is still limited. In the present study, biofilm formation of the *Staphylococcus aureus* ATCC 25923 strain was studied on clinically relevant materials—borosilicate glass, plexiglass, hydroxyapatite, titanium and polystyrene—at 18, 42 and 66 h. Materials with the highest surface roughness and porosity (hydroxyapatite and plexiglass) did not promote biofilm formation as efficiently as some other selected materials. Matrix-associated poly-*N*-acetyl-β-(1-6)-glucosamine (PNAG) was considered important in young (18 h) biofilms, whereas proteins appeared to play a more important role at later stages of biofilm development. A total of 460 proteins were identified from biofilm matrices formed on the indicated materials and time points—from which, 66 proteins were proposed to form the core surfaceome. At 18 h, the appearance of several r-proteins and glycolytic adhesive moonlighters, possibly via an autolysin (AtlA)-mediated release, was demonstrated in all materials, whereas classical surface adhesins, resistance- and virulence-associated proteins displayed greater variation in their abundances depending on the used material. Hydroxyapatite-associated biofilms were more susceptible to antibiotics than biofilms formed on titanium, but no clear correlation between the tolerance and biofilm age was observed. Thus, other factors, possibly the adhesive moonlighters, could have contributed to the observed chemotolerant phenotype. In addition, a protein-dependent matrix network was observed to be already well-established at the 18 h time point. To the best of our knowledge, this is among the first studies shedding light into matrix-associated surfaceomes of *S. aureus* biofilms grown on different clinically relevant materials and at different time points.

## 1. Introduction

Implanted medical devices have been consistently shown to improve the quality of life of patients suffering from critical conditions such as the destruction of joints [1]. Increasing life span and evolving medical sciences have accelerated the use of such devices. However, paradoxically, this trend has also increased the propensity to device-associated infections. In joint replacements, infection rates have been estimated to be approximately 1.7% in hip, 2% in knee and 9% in ankle prostheses [2]. In addition, 5–10% of inserted internal fixation devices can typically become infected [3]. Elderly, obese, malnourished, diabetic and rheumatic patients and near-surface body inserts with poor soft tissue coverage are most vulnerable to such infections. Medical device-associated infections are common complications of implantation surgery caused by material-colonizing microbial communities, also known as biofilms [1].

*Staphylococcal* spp. biofilms are one of the frequent causes of certain medical device-associated infections, such as infections related to intravenous catheters [4,5], cardiac pacemakers [6] and mammary implants [7,8]. In prosthetic joint infections, especially *Staphylococcus aureus* is largely responsible for early post-interventional infection types [9]. Biofilms refer to complex communities of bacteria, which can be attached to a surface or form aggregates without attachment to any surface. Biofilms differ from free-floating cells by slow growth and tolerance to antibiotics and immune cells of the host [10,11,12,13]. Freely floating bacteria are responsible for colonizing new niches, while the biofilm lifestyle, preferred by most of the bacteria, serves as a survival strategy against external threats [14]. Increased adaptation, adherence and antibiotic tolerance are characteristic features of the bacterial biofilm. These features are to a great extent connected to a slimy structure surrounding the bacterial cell community known as the biofilm matrix, which forms approximately 90% of the biofilm dry mass and it is comprised of hydrated extracellular polymeric components (EPS), such as exopolysaccharides, proteins, lipids and nucleic acids [15]. The biofilm matrix is likely to change as biofilms develop over time, and it is also different between biofilms formed on different surfaces. Those changes in the biofilm matrix are likely crucial for understanding antibiotic tolerance and designing better anti-biofilm therapies. However, the dynamics of such changes have been poorly investigated, thus far.

Bacteria may colonize indwelling medical devices during implantation or at later stages by hematogenous seeding. After implantation, medical devices are promptly coated by host proteins, involving fibrinogen, fibronectin and fibrin, which can facilitate adhesion of bacteria like *Staphylococcus aureus* that express receptors for binding these plasma proteins [16,17]. The implant-induced changes in the host, such as reduced blood flow and impaired activity of lymphocytic and phagocytic cells, are other factors that can also promote the biofilm formation on medical devices [1]. Besides, some substrate materials such as polymethyl methacrylate, acrylic glass (PMMA) can intrinsically contribute to biofilm formation by hampering the complement and reducing leukocytic activity. Notably, phagocytic cells often focus on degrading the medical device itself, not the bacteria. Strategies to cope with such infections have involved systemically administered antibiotics [18]. However, achieving an adequate concentration of the antibiotic at the biofilm infection site is difficult due to reduced local tissue perfusion and compromised vasculature [19,20]. Thus, removal of the device and debridement of the necrotic tissue is often required in conjunction with the insertion of a new medical device, which often leads to revision surgeries and prolonged hospitalization periods with a high risk for re-infections [3,21]. A systemic antibiotic prophylaxis during the time of the surgery together with the coating of the inserted devices have been suggested among the strategies to improve clinical outcomes with implanted medical devices [18].

A wide range of different implant materials are used in orthopedics. Despite the intensive research currently being performed on different technologies for incorporating antimicrobial agents [22,23,24,25,26,27,28,29,30,31,32], only a few studies investigating biofilm formation on different clinically relevant implantation materials have been published [33,34,35,36,37,38]. The dynamic changes associated with biofilm growth [39] make biofilm eradication from clinical materials even more challenging. As structural and functional features of biofilms are greatly dependent on the material and the biofilm growth conditions [38,40,41], there is an obvious demand for comprehensive studies investigating the structural and functional features of the prosthetic materials together with biofilm dynamics on these materials. In this context, the cell surface-associated adhesins have attracted great interest, as many of these can contribute to protein-mediated biofilm formation [39,42,43,44,45,46]. Recently, an unacknowledged group of surface-associated proteins, i.a., moonlighting virulence factors and cytoplasmic proteins embedded in the staphylococcal biofilm matrix, was proposed to form a new molecular mechanism conferring increased stability for biofilm population [47]. High levels of intracellular cytoplasmic proteins and, to a much lesser extent, extracellular or cell-surface adhesins have also been identified from in vivo biofilm matrices using a rat model of orthopedic implant-associated chronic *S. aureus* infection [48]. Whether these moonlighters play a role during the formation of *S. aureus* biofilms on different prosthetic materials is not yet clear.

Thus, the aim of the present study was to compare the biofilm characteristics of a *S. aureus* biofilm-forming model strain ATCC 25923 (also known as strain Rosenbach or Seattle 1945) [49] on different clinically pertinent substrates, involving borosilicate glass, plexiglass, hydroxyapatite and titanium, using polystyrene as the reference material. Borosilicate glass (G) has been traditionally used in artificial hip joints, bone cements, dental composite materials, prosthetic eyes and breast implants [50,51], while plexiglass (PG) is used in bone cements [52]. Joint implants are made of titanium alloys (TI) due to their biocompatibility and corrosion resistance, while hydroxyapatite (HA), a known mineral component of bones, is used to coat medical devices for improving the integration of the implant with newly formed bone [53]. The present study focused on (1) comparing the susceptibility of these clinically relevant surfaces to biofilm formation, (2) studying the time-dependent variations in exopolysaccharide and protein compositions of the biofilms on the indicated materials, and (3) identifying the core surfaceome (proteins present in all aged biofilms (18, 42, and 66 h) on every material) contributing to adherence/adherent growth. The present study demonstrates a number of material- and time point-dependent surfaceome changes. As a main finding, matrix-associated poly-*N*-acetyl-β-(1-6)-glucosamine (PNAG) was regarded as important in young (18 h) biofilms, whereas proteins had a more pivotal role at later stages of biofilm development.

## 2. Materials and Methods

### 2.1. Materials

Tryptic soy agar (TSA) and tryptic soy broth (TSB) were acquired from Lab M Limited (Lancashire, UK), while sucrose was from Merck (Darmstadt, Germany). Sequencing-grade modified trypsin (porcine) was purchased from Promega Corp. (Madison, WI, USA). Wheat germ agglutinin Alexa Fluor^®^ 488 conjugate (WGA) was obtained from Invitrogen™, Thermo Fisher Scientific (Eugene, OR, USA). Dimethyl sulphoxide (DMSO) was purchased from VWR International (Fontenay-sous-Bois, France) and phosphate-buffered saline (PBS) was supplied by Lonza (Verviers, Belgium). Trifluoroacetic acid (99%), triethylammonium bicarbonate buffer (TEAB; 1.0 M, pH 8.5), Tween^®^ 20, vancomycin hydrochloride hydrate, penicillin G sodium salt, levofloxacin and doxycycline hydrochloride were purchased from Sigma-Aldrich (Steinheim, Germany).

### 2.2. Atomic Force Microscopy (AFM)

The topography of the material surfaces (borosilicate glass, G; plexiglass, PG; hydroxyapatite, HA; titanium, TI and polystyrene, PS) was characterized with an NTegra Prima AFM (NT-MDT, Moscow, Russia) in an intermittent contact mode by using Au-coated NSG10 (NT-MDT, Moscow, Russia) probes with a nominal tip curvature radius of 10 nm and a force constant of 3.1–37.6 N/m. A scan rate of 0.3–0.5 Hz was used. Image analysis was conducted by using the Scanning Probe Image Processor software (SPIP, Image Metrology, Hørsholm, Denmark). Captured topographs were processed with Gaussian (ISO 11562) filtering.

### 2.3. Bacterial Culture and Biofilm Formation

The clinical strain *Staphylococcus aureus* ATCC 25923 was cultured in TSB at 37 °C under aerobic conditions with shaking (220 rpm) to reach the exponential phase, to a concentration of 10^8^ CFU·mL^−1^. The bacterial concentration was estimated by measuring optical density at 595 nm (Varioskan™ LUX Multimode Microplate Reader, Thermo Scientific, Vantaa, Finland) and later confirmed by calculating colony-forming units (CFUs) on TSA plates. Biofilms were formed on 96-well plates (96-WPs) (Nunclon™ Δ surface polystyrene plates, Nunc™, Roskilde, Denmark) or on coupons made of borosilicate glass (G), plexiglass (PG), titanium (TI) with dimensions: 0.4 cm height, 1.27 cm diameter; and hydroxyapatite (HA) with dimensions: 0.25 cm height, 1.27 cm diameter (BioSurface Technologies Corporation, Bozeman, MT, USA). For the experiments with coupons, they were placed in a polystyrene 12-WP (Costar^®^, flat bottom, well diameter 2.26 cm; Corning Inc., Corning, NY, USA). For the experiments performed in 12-WPs (with the coupons on them) or 96-WPs, 2.5 mL or 200 µL of the bacterial suspension (10^6^ CFU·mL^−1^) were used, respectively. The plates were incubated at 37 °C under aerobic conditions with shaking (150 rpm) for 18, 42 or 66 h (the incubation times were based on [54]). The media of the 42-h-old biofilms were changed after 18 h of incubation, whereas the media of the 66-h-old biofilms were refreshed at 18 and 42 h.

### 2.4. Quantification of Biofilm Formation on Different Materials

*S. aureus* ATCC 25923 was grown on polystyrene (PS) (in 96-WP) or on borosilicate glass (G), plexiglass (PG), hydroxyapatite (HA) and titanium (TI) coupons in 12-WP, as described above. After 18, 42 or 66 h, the biofilms formed on G, PG, HA or TI coupons were disaggregated for quantification as follows. First, the coupons were soaked in the medium (TSB) to detach planktonic and loosely attached bacteria, and then transferred into Falcon tubes of 50 mL containing 1 mL of 0.5% (*w/v*) Tween^®^ 20-TSB solution. In contrast, biofilms grown on 96-WPs were washed once with 200 µL of TSB, and 200 µL of Tween^®^ 20-TSB solution was added on wells. The tubes and the 96-WPs were sonicated in a water bath in Ultrasonic Cleaner 3800 (Branson Ultrasonics, Danbury, CT, USA) at 25 °C, 35 kHz, for 5 min. Serial dilutions (10^−1^–10^−8^) were performed from the resulting bacterial suspensions onto TSA plates. Size differences between coupons and 96-WPs were taken into account by measuring colony-forming units (CFU) per volume (mL) and area of bacterial attachment on the different surfaces (cm^2^) and transforming the values of CFU·(mL·cm^2^)^−1^ to a log_10_ scale.

### 2.5. Quantification of Matrix-Associated Poly-N-Acetyl-β-(1-6)-Glucosamine

For quantifying matrix poly-*N*-acetyl-β-(1-6)-glucosamine (PNAG), *S. aureus* ATCC 25923 biofilms were grown in 96-WPs and on coupons in 12-WPs for 18, 42 and 66 h, as described above. The previously reported WGA staining protocol [54] was applied with two modifications: a lower concentration (2.5 µg·mL^−1^) of WGA conjugate was used based on [55] and 100% DMSO was used to replace 33% acetic acid, which is not compatible with acid-unbearable materials, such as HA. The selection of 100% DMSO was based upon initial tests where a lower concentration of acetic acid (10% acetic acid), 96% ethanol and 100% DMSO were tested (acquired assay quality parameters for DMSO: screening window coefficient Z’ factor = 0.431; signal to noise (S/N) = 5.4; signal to background (S/B) = 9.2)

First, the 96-WPs were washed once with 200 µL of PBS, while the coupons were briefly soaked once in PBS, to detach planktonic and loosely attached cells. Then, WGA in PBS (2.5 µg·mL^−1^) was added onto 96-WPs (200 µL) or onto coupons (500 µL) in 24-WPs (Nunclon™ Δ surface, Nunc™, Roskilde, Denmark) and incubated in the darkness at 4 °C for 2 h. After the incubation, the biofilms were washed three times with PBS and dried at room temperature (RT) for 15 min. Next, 200 µL of DMSO was added into the 96-WPs. The coupons were transferred into Falcon tubes of 50 mL, containing 1.3 mL (for G, PG and TI) or 1.11 mL (for HA coupons; smaller volume was due to the smaller size of these coupons) of DMSO. The plates and the tubes were sonicated in a water bath at 25 °C, 35 kHz, for 30 s. Following an incubation at 37 °C for 1 h, the sonication step was repeated. Finally, 200 µL of the remaining suspensions from Falcon tubes were transferred onto a 96-WP to measure top fluorescence with Varioskan™ LUX Multimode Microplate Reader (λ_excitation_ = 495 nm; λ_emission_ = 520 nm). Before measuring the fluorescence signal from biofilms formed on 96-WPs, the resulting suspensions were diluted 1:10 to be in correspondence with the coupon surface area.

### 2.6. Fluorescence Imaging

These experiments were performed with *S. aureus* ATCC 25923 biofilms grown on G coupons, stained with WGA as indicated above. After the 2-h-long incubation period (4 °C, in the darkness), the unbound dye was removed, and images of the coupons on Petri dishes were acquired with an Invitrogen™ EVOS^®^ FL Imaging System (Life Technologies™, Eugene, OR, USA) using the Green Fluorescent Protein (GFP) light cube (λ_excitation_ = 470/22 nm; λ_emission_ = 510/42 nm) and a 20× objective.

### 2.7. Trypsin Shaving of Matrix-Associated Proteins

Before protein identification, biofilm viability with and without the trypsin treatment was first assessed to exclude possible effects of trypsin on biofilm integrity. Biofilms were grown on G coupons in 12-WPs and then transferred into Falcon tubes (50 mL) containing 1 mL of 100 mM acetate buffer (pH 4.7), a condition preventing the release of the adhesive moonlighting proteins [56]. The tubes were sonicated (25 °C, 35 kHz, 5 min) and detached bacterial cells were collected by centrifugation (4 °C, 4000× *g*, 2 min). The cells were suspended in 110 μL of 100 mM TEAB containing 16% sucrose (TEAB-sucrose (16%); pH 8.5) with and without trypsin (at a final concentration of 51.9 ng·µL^−1^), and the mixtures were incubated (37 °C, 15 min). Next, the cells were serially diluted in TSB and plated onto TSA to determine CFUs. The number of viable counts remained the same in both samples, excluding the possibility of trypsin-induced cell lysis (Figure S1) and confirming the suitability of the shaving conditions for trypsin shaving.

Biofilm cells for trypsin shaving were prepared in duplicates for each substrate and time point as follows. Biofilms attached to coupons were first rinsed with TSB to remove planktonic bacteria and transferred onto 12-WPs. There, the cells were scraped off from the coupon surface into 100 mM acetate buffer (4 °C, pH 4.7) using a sterile plastic stick. In the case of biofilms formed on PS, the biofilms were also rinsed with TSB first and scraped off from 96-WPs into 100 mM acetate buffer. In all cases, cells were collected by centrifugation (4 °C, 4000× *g*, 2 min) and suspended in 110 μL of 100 mM TEAB-sucrose (16%) [57] and trypsin (at a final concentration of 51.9 ng·µL^−1^). After the trypsin treatment (37 °C, 15 min), cells were first removed by centrifugation (RT, 4000× *g*, 2 min) and digestions were further purified through cellulose acetate membranes (pore size 0.22 μm, Costar^®^ Spin-X Centrifuge Tube Filter, Corning Inc., Corning, NY, USA) by centrifugation (RT, 16000× *g*, 2 min). Digestions incubated at 37 °C for 16 h were blocked by adding trifluoroacetic acid to a final concentration of 0.6%. Concentrations of released proteins/peptides were measured using low-volume photometric quantification at 280 nm, with a µDrop™ Plate (Thermo Scientific, Vantaa, Finland) on a Varioskan™ LUX Multimode Reader (Thermo Scientific, Vantaa, Finland).

### 2.8. Identification of Trypsin-Released Proteins/Peptides by LC–MS/MS

Tryptic peptides were purified and concentrated using ZipTips (C18; Millipore^®^, Merck KGaA, Darmstadt, Germany) and peptides were analyzed essentially as described previously [58]. Briefly, an equal amount of the purified tryptic peptides was submitted to an Easy-nLC 1000 Nano-LC system (Thermo Scientific, Vantaa, Finland) coupled to a quadrupole Orbitrap mass spectrometer (Q Exactive™, Thermo Scientific, Bremen, Germany) equipped with a nanoelectrospray ion source (Easy-Spray™, Thermo Scientific, Vantaa, Finland). Liquid chromatography separation was performed in an Easy-Spray™ column of 25 cm bed length (C18, 2 μm beads, 100 Å, 75 μm inner diameter, Thermo Scientific), using a flow rate of 300 nL/min. The peptides were eluted with a 2–30% gradient of solvent (composed of 100% acetonitrile and 0.1% formic acid) in 60 min. Acquired MS raw data were processed using the MaxQuant version 1.6.2.1 [59] with built-in Andromeda search engine [60]. Database searches were conducted against the UniProt *S. aureus* protein database (https://www.uniprot.org/). In these searches, carbamidomethyl (C) was set as a fixed and methionine oxidation as a variable modification. First search peptide tolerance of 20 ppm and main search error of 4.5 ppm were used. Trypsin without proline restriction enzyme option was used, with two allowed miscleavages. The minimal unique + razor peptides number was set to 1, and FDR to 0.01 (1%) for peptide and protein identification. Known contaminants provided by MaxQuant, and proteins identified as “reverse” and “only identified by site” were discarded from further data analyses. Only proteins that could be identified in both replica samples were included in data set comparisons.

### 2.9. Chemotolerance Assays

*S. aureus* ATCC 25923 biofilms were grown on HA and TI coupons in 12-WPs for 18 and 66 h, as previously described in Section 2.3. After the incubation, the chemotolerance assay was performed essentially as described previously [28]. The coupons were soaked in TSB to detach planktonic cells and transferred onto 12-WPs containing 2.5 mL of 2.0 µM penicillin G (0.71 µg/mL), 90.0 µM levofloxacin (32.5 µg/mL), 4.0 µM doxycycline (1.92 µg/mL) or 5.0 µM vancomycin (7.43 µg/mL) or TSB (as a negative control). The coupons were exposed to the indicated antibiotics for 2 or 24 h at 37 °C, under aerobic conditions with shaking (150 rpm). After the treatment, biofilms were quantified as in Section 2.4. A combination treatment involving trypsin (51.9 ng·µL^−1^) and 90 µM levofloxacin was also tested. Therein, biofilms were formed on HA for 18 and 66 h, as above. After the incubation periods, the coupons were soaked in TSB and transferred onto a 24-WP containing trypsin in buffer (35 µL of trypsin in 350 µL of 100 mM TEAB-sucrose (16%)) or mere buffer (385 µL of 100 mM TEAB-sucrose (16%), as a negative control). The 24-WPs were incubated at 37 °C for 15 min, as described in Section 2.7. Next, the coupons were soaked in TSB, and transferred onto the 12-WP containing 2.5 mL of 90 µM levofloxacin. The coupons were incubated with the antibiotic at 37 °C under aerobic conditions with shaking (150 rpm) for 24 h. As a second control, biofilms were grown in TSB under similar conditions for 24 h and 15 min. Biofilm formation on the materials was assessed as in Section 2.4. The anti-biofilm effect of the antibiotics is expressed as a logarithmic reduction of the bacterial burden [61], using Equation (1):

(1) $$logR\;=log_{10}\langle {\left(CFU/mL\right)}_{control}\rangle -log_{10}\langle {\left(CFU/mL\right)}_{compound}\rangle$$

where 〈·〉 denotes averaging over samples.

### 2.10. Data Processing and Statistical Analysis

For the optimization of the WGA staining protocol (Section 2.5), the following statistical parameters were used: screening window coefficient *Z*′ factor, signal to noise (*S*/*N*) and signal to background (*S*/*B*) (according to Equations (2)–(4)) [54,62,63].

(2) $$Z^{\prime }=1-\frac{\left(3\;x\;SD_{max}+3\;x\;SD_{min}\right)}{\left|X_{max}-X_{min}\right|}$$

(3) $$\frac{S}{N}=\frac{X_{max}-X_{min}}{{\left({SD_{max}}^{2}+{SD_{min}}^{2}\right)}^{1/2}}$$

(4) $$\frac{S}{B}=\frac{X_{max}}{X_{min}}$$

One-way analysis of variance comparisons and Tukey (for equal variances) and Games–Howell (for unequal variances) post-tests were executed using IBM SPSS Statistics (SPSS Inc., Chicago, IL, USA, version 24.0 for Windows). In paired comparisons, the unpaired t-test with Welch′s correction was used (GraphPad Software, Prism, La Jolla, CA, USA, version 7.0 for Windows). *p* < 0.05 was considered statistically significant and *p* < 0.001 statistically highly significant. Each test was performed at least in duplicate.

SPSS was also used in multivariate statistical analyses of the protein identification data, using average relative intensity values obtained for proteins identified in both biological replicates. The values of the commonly identified proteins were log_2_-transformed, and principle component analysis (PCA; based on the correlation matrix) was performed using Oblimin rotation with Kaiser Normalization.

## 3. Results

### 3.1. HA and PG Exhibited the Largest Surface Roughness

**Figure 1:**
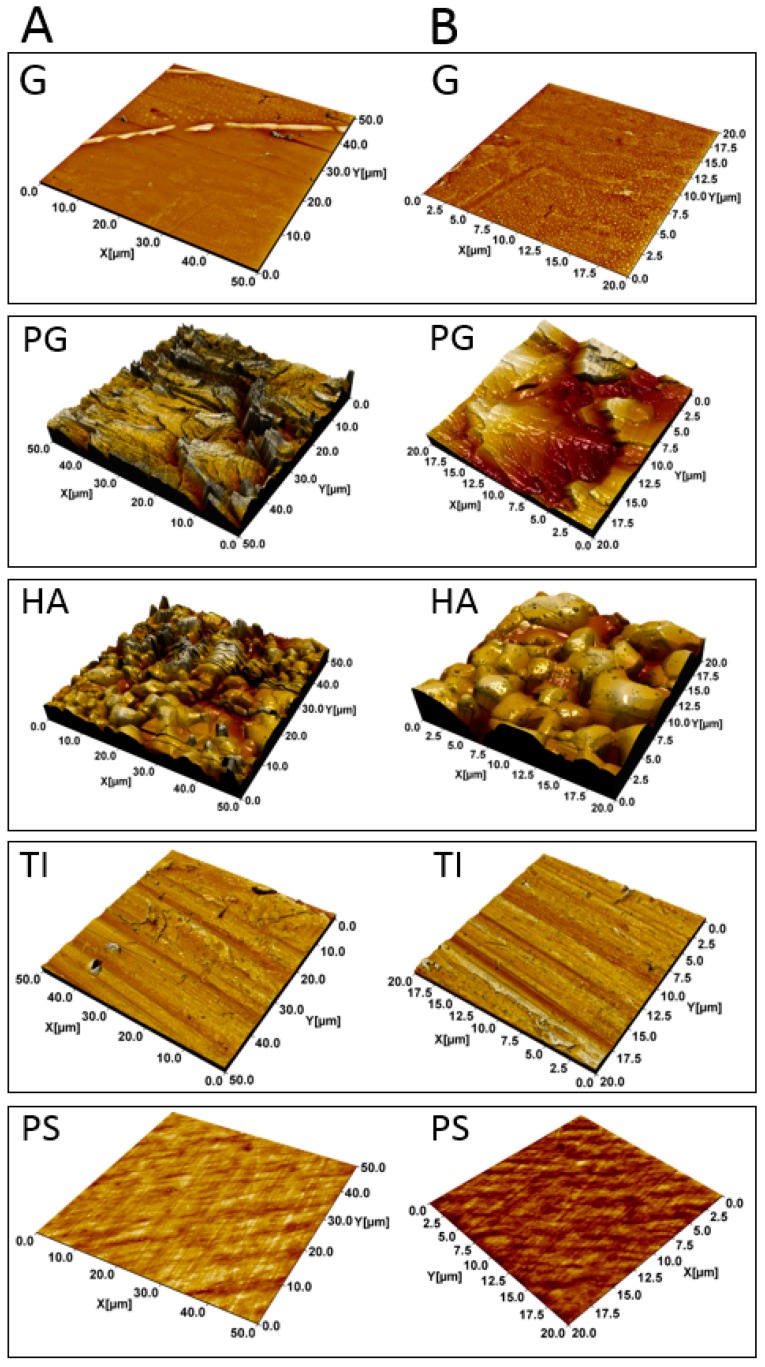
Atomic Force Microscopy (AFM) topographical images of the materials. Borosilicate glass (G), plexiglass (PG), hydroxyapatite (HA), titanium (TI) and polystyrene (PS) captured with the image size of 50 × 50 µm (**A**). Zoomed images of the materials with the size of 20 × 20 µm (**B**).

AFM topographical images of 96-well plates (made of polystyrene; PS) and borosilicate glass (G), plexiglass (PG), hydroxyapatite (HA) and titanium (TI) coupons are shown in Figure 1. Materials PG and HA are the most heterogeneous and uneven surfaces, whereas TI, PS and G appear to be much smoother. Roughness analysis of the AFM images provided more quantitative insights into the differences between the substrates (Table 1 and Figure S2). The surface area ratio (S_dr_) describes the roughness-induced increment of the interfacial surface area relative to the area of the projected flat plane, while V_v_ illustrates the void volume, describing surface porosity. The surfaces of HA and PG showed the largest roughness values (S_dr_ = 58 ± 10% and 123 ± 20%; V_v_ = 0.75 ± 0.08 µm^3^/µm^2^ and 0.62 ± 0.07 µm^3^/µm^2^, respectively) (Table 1). The smoothest surface was observed to be G (S_dr_ = 0.3 ± 0.1%; V_v_ = 0.0048 ± 0.001 µm^3^/µm^2^). The same trend was observed with length-scale dependent roughness (Figure S2).

### 3.2. The Most Significant Time-Dependent Increase in Biofilm Formation Was Detected on HA

**Figure 2:**
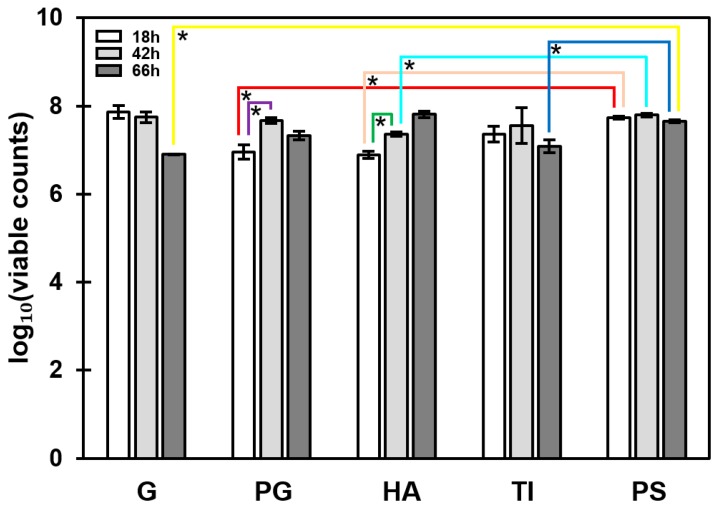
Formation of *Staphylococcus aureus* ATCC 25923 on five substrate materials. Bacterial attachment is expressed as log_10_ values of CFU·(mL·cm^2^)^−1^. Differences in attachment were assessed for a single material between different time points, and for a fixed time point between the different materials, using one-way ANOVA with Games–Howell post-test. *, significant difference (*p* < 0.05). Error bars denote the standard error of the mean (SEM) (*n* = 3). G, borosilicate glass; PG, plexiglass; HA, hydroxyapatite; TI, titanium; PS, polystyrene.

Attachment of *S. aureus* ATCC 25923 onto all five materials was compared at three incubation time points (18, 42 and 66 h). The number of attached bacteria was expressed as log_10_ of viable CFU·(mL·cm^2^)^−1^. In the assay conditions, viable colonies increased temporally in a statistically significant manner only in two cases (PG and HA; from 18 to 42 h), while a non-significant trend implying temporal decrease in cell viability was observed with biofilms on G and TI (Figure 2). Comparisons between different materials at similar time points showed that *S. aureus* ATCC 25923 was more prone to form biofilms on PS than on the other substrates. In addition, biofilm formation on PS was equally high after 18, 42 or 66 h.

### 3.3. Temporal Decrease in the Total PNAG Amount Was Detected in All Biofilms

**Figure 3:**
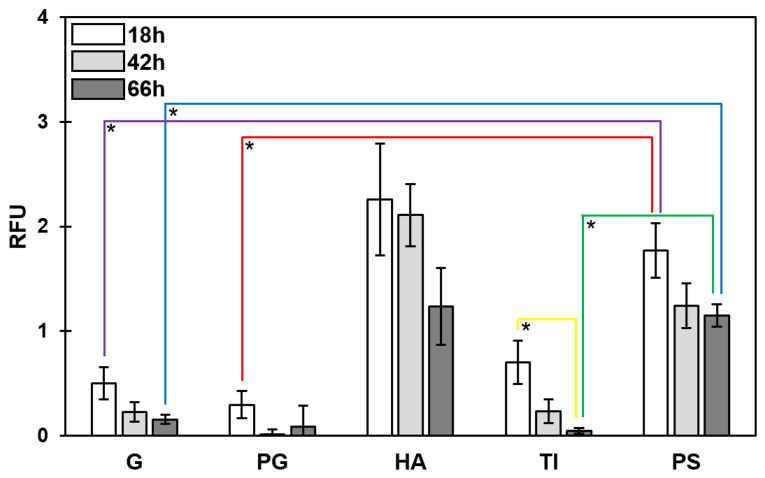
Wheat germ agglutinin Alexa Fluor^®^ 488 conjugate-based quantification of poly-*N*-acetyl-β-(1-6)-glucosamine (PNAG) in *S. aureus* ATCC 25923. Differences in PNAG contents (expressed as relative fluorescence units (RFUs)) were assessed for a single material between different time points, and for a fixed time point between the different materials, using one-way ANOVA comparisons and Games–Howell post-tests for blank-corrected data points. *, significant difference (*p* < 0.05). Error bars denote the standard error of the mean (SEM) (*n* = 2). G, borosilicate glass; PG, plexiglass; HA, hydroxyapatite; TI, titanium; PS, polystyrene.

**Figure 4:**
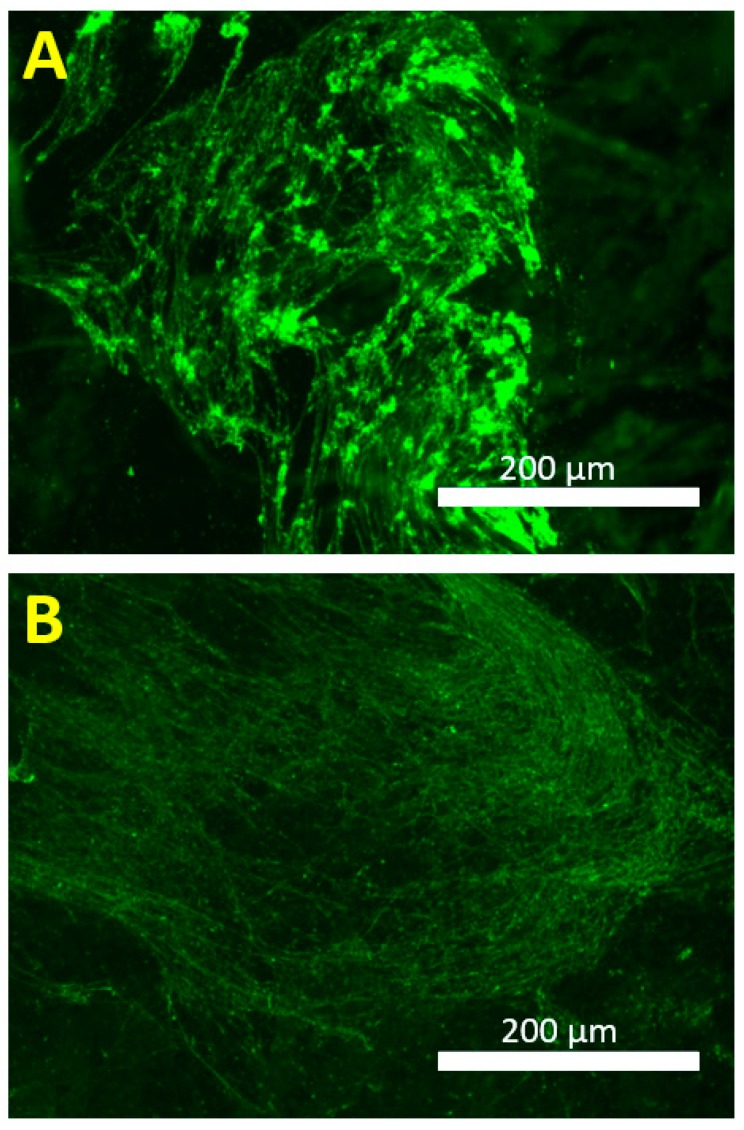
WGA-Alexa Fluor^®^ 488 fluorescent conjugate-based imaging of the 18- (**A**) and 42-h-old (**B**) *S. aureus* ATCC 25923 biofilm matrix. Biofilms were formed on borosilicate glass coupons and the images were taken using Invitrogen™ EVOS^®^ FL Imaging System.

The exopolysaccharide amount was studied using a wheat germ agglutinin (WGA) conjugate that targets the poly-*N*-acetyl-β-(1-6)-glucosamine (PNAG) fraction of the biofilm matrix. Using the optimized staining conditions (described in Section 2.5), 18-, 42- and 66-h-old *S. aureus* ATCC 25923 biofilms were treated with WGA conjugate, after being formed on the indicated five substrate materials. The PS-associated biofilms contained statistically more (*p* < 0.05) PNAG fraction than biofilms formed on G, PG or TI (Figure 3). A temporal decline (from 18 to 66 h) in the PNAG fraction was noted for all the tested substrate materials; the deepest decline was detected with biofilms on TI. The images presented in Figure 4 confirm the declining trend for PNAG in 18- versus 42-h-old biofilms on G coupons, and illustrate the macrostructural temporal evolution from thick, intermittent regions (Figure 4A; 18 h) to a thinner, more cohesive PNAG network (Figure 4B; 42 h).

### 3.4. A Total of 66 Proteins Were Shared by All Biofilms

**Figure 5:**
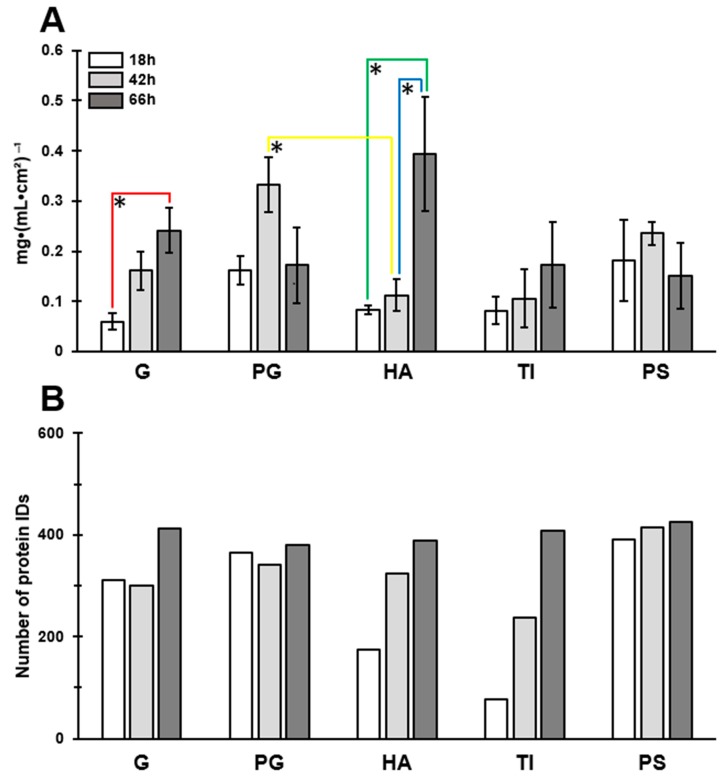
Protein concentrations mg·(mL·cm^2^)^−1^ (**A**) and the number of the identified matrix-associated proteins (detected in both replicates) (**B**) of the *S. aureus* ATCC 25923 biofilms. Differences in protein concentrations were assessed for a single material between different time points, and for a fixed time point between the different materials, using one-way ANOVA comparisons and Tukey post-tests for blank-corrected data points. *, significant difference (*p* < 0.05). Error bars denote the standard error of the mean (SEM) (*n* = 2). G, borosilicate glass; PG, plexiglass; HA, hydroxyapatite; TI, titanium; PS, polystyrene.

The protein concentration and the number of proteins were first examined in all biofilm matrices at the indicated time points (18, 42 and 66 h). The clearest trend in time-dependent increase in the total protein concentration was detected for biofilms formed on G and HA (Figure 5A). For the PG- and PS-associated biofilms, the highest protein concentration was reached at the 42 h time point, although later declined at 66 h in both cases.

**Figure 6:**
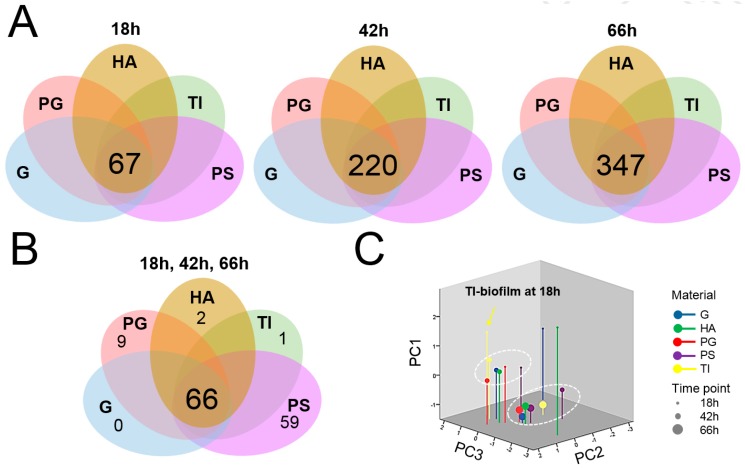
Venn diagrams representing the shared surfaceome proteins present on all materials at fixed time points of growth (**A**) and the number of all identified proteins shared by each biofilm and material combination and time point (**B**). A three-dimensional principle component analysis (3D PCA) analysis indicating outliers and clusters (circled) nested within the identified material- and time-dependent biofilm surfaceomes (**C**).

Next, all the individual proteins were identified from the biofilm samples by trypsin shaving and LC–MS/MS analyses. The combined surfaceome catalogs based on two independent experiments enabled the identification of 460 proteins in total. An obvious trend of temporal increase in the number of the identified proteins was observed with biofilms formed on HA and TI (Figure 5B). At the 18 h time point, the highest number of proteins was identified from biofilms formed on PS (390 proteins), and the lowest number with the biofilms formed on TI (76 proteins). The core surfaceomes (i.a., protein orthologs shared by all samples) defined for biofilms growing on different materials at a fixed time point indicated 67, 220 and 347 proteins that were shared by all 18, 42 and 66 h time point biofilms, respectively (Figure 6A). The number of proteins common to all biofilms (detected on every material at every time point) was 66 (Figure 6B). The highest number of proteins shared by all time points in fixed materials was obtained with the PG and PS biofilms (PG, 329 proteins; PS, 383 proteins) (Figure S3).

### 3.5. Protein Moonlighters Formed the Largest Fraction of the Core Surfaceome

Dynamics of relative protein abundance changes in each formed biofilm over time was investigated next by three-dimensional principal component analysis (3D PCA). Figure 6C shows two major clusters; the 42 h PS biofilm surfaceome together with those formed on each five materials for 66 h, while the rest of the 42 h biofilm surfaceomes (G, HA, PG and TI) and those associated with PG- and PS at the 18 h time point formed the second cluster. Biofilms formed on TI-, G- and HA-coupons at the 18 h time point were clearly separated from the two main clusters, implying that these three materials affected the adherence in young biofilms (during 18 h). Altogether, the 3D PCA analysis revealed that TI, G and HA promoted specific surfaceome changes in biofilms already at the 18 h time point.

Table 2 illustrates changes in protein abundances in relation to time and different substrates. The most dominating proteins in all materials and time points were identified either as known or potential moonlighting proteins. Among these, the ribosomal proteins (r-proteins) formed the biggest group. Other moonlighters identified in all materials and time points included enzymes with a role in glycolysis (enolase (ENO); glyceraldehyde-3-phosphate dehydrogenase (GaPDH); triosephosphate isomerase (TPI); pyruvate kinase (PYK); pyruvate dehydrogenase E1 (PDHB); phosphoglycerate kinase (PGK); l-lactate dehydrogenase 1 (l-LDH) and alcohol dehydrogenase (ADH)), protein synthesis (elongation factor Tu (EfTU); elongation factor G (EfG) and elongation factor P (EfP)), and stress (chaperone protein (DnaK); universal stress protein (SAV1710), Usp and alkyl hydroperoxide reductase (AhpC)). Among these, the predicted abundances of EfTU, PGK, ENO and GaPDH reached the highest level at 66 h, which clearly exceeded those detected for the r-proteins. The Clp family proteins (ClpP, ClpL, ClpC and ClpB) also displayed material-dependent variation; their abundances displayed temporal increase in all biofilm matrices. The Clp proteins were not detected at the 18 h time point of TI.

A high number of different virulence factors and factors conferring increased resistance to one or several antibiotics were also detected (Table 2). From these, gamma-hemolysin component B (HlgB), leukocidin-like proteins (Luk1/2), IgG-binding proteins (Sbi and Spa) and immunodominant antigens (IsaA and IsaB) were among the high abundant proteins in all materials at each time point. Several regulatory proteins were present in one or several of the materials already in young biofilms—among these, the relative abundance of CcpA, CodY, SarR, SaeR, Rot, MsrR, Rex and VraR increased over time, reaching the highest level at the 66 h time point. Enzymes involved in maintaining the cellular redox state (e.g., peptide methionine sulfoxide reductase (MsrB)) were either present or absent at 18 h in biofilms but could be detected with higher identification scores in all biofilms at the 42 and 66 h time points (Tables S1 and S2).

### 3.6. Greatest Time-Dependent Variations Were Observed for TI- and HA-Associated Surfaceomes

Comparing the number of uniquely identified proteins at different time points of growth revealed the greatest differences in TI- and HA-associated surfaceomes (Figure S3). These surfaceomes displayed a relatively high number of specific proteins at 66 h (HA, 74 proteins; TI, 169 proteins) and proteins shared by these two matrices at the 42 and 66 h time points (HA, 142 proteins; TI, 164 proteins) could not be identified in other biofilm matrices. Of note, at the 18 h time point, TI-associated biofilm cell surfaces were devoid of several proteins that were identified from other biofilms at this time point.

The classically secreted cell wall/membrane-anchored adhesins were either present or absent in one or several of the investigated biofilms at the 18 h time point (Table 2). These included the fibronectin (FnBPA) and fibrinogen binding proteins (FbnBP). For instance, FnBPA was not detected in PS, HA or TI biofilms, while HA biofilms lacked the FbnBP adhesion at this growth stage (18 h). The bone sialoprotein-binding protein (Bbp) was not present in G and TI biofilms (18 h), while elastin binding proteins S (EbpS) was detected only in PS and G biofilms. The clumping factors A and B (ClfA, ClfB) were also differently abundant on the tested materials at the 18 h time point: ClfA was specific to PS and PG biofilms, while ClfB was detected on every material except on TI. On the other hand, adhesins that were not detected at the 18 h time point (or detected with lower abundances) could be identified with reasonably high identification scores at the later time points of growth (42- and/or 66 h). Such proteins included Bbp, FbnBP and the clumping factors ClfA and ClfB. In addition, FnBPA was not identified in any biofilm surfaceomes at the later time point (66 h). Glutamine synthetase, a potential moonlighting adhesin, was specifically identified only in TI biofilms at each time point of growth.

### 3.7. Antibiotic Susceptibility Depends on the Composition of the Biofilm Surfaceome

**Figure 7:**
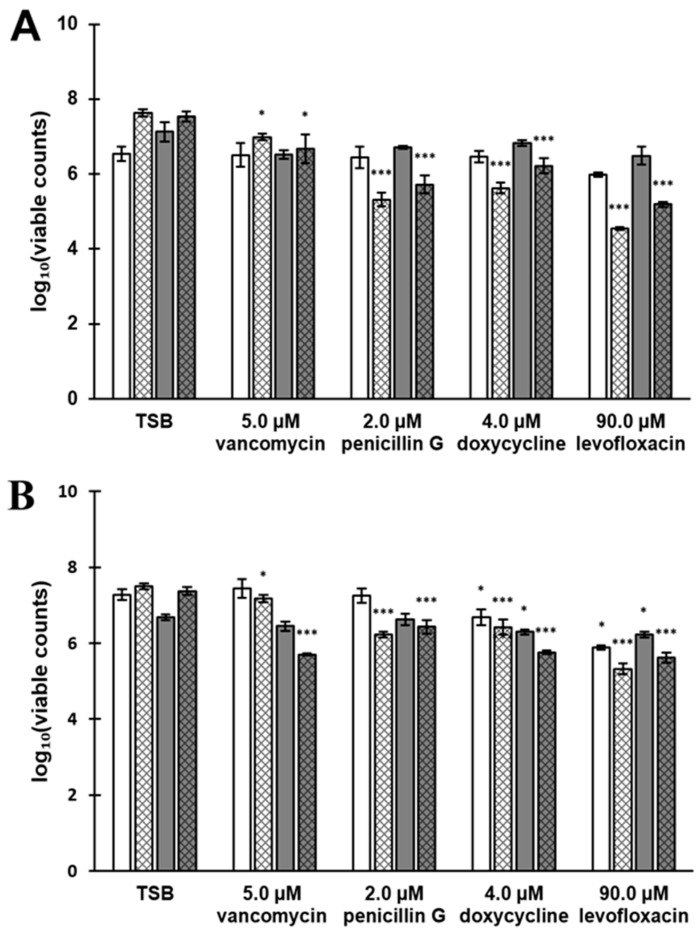
The chemotolerance of 18- and 66-h-old *S. aureus* ATCC 25923 biofilms formed on hydroxyapatite, HA (**A**) and titanium, TI (**B**) and exposed to several antibiotics for 2 or 24 h. The results are expressed as log_10_ values of CFU·(mL·cm^2^)^−1^. Plain white and dark grey bars illustrate 18- and 66-h-old biofilms (respectively) with 2-h-long antibiotic exposure, while striped white and dark grey bars illustrate 18- and 66-h-old biofilms (respectively) with 24-h-long antibiotic exposure. The results of antibiotic-treated biofilms were compared to TSB-treated biofilms. The statistical analysis was performed by using unpaired t-tests with Welch’s correction. * *p* < 0.05 and *** *p* < 0.001 were considered statistically significant and highly significant, respectively. Error bars denote the standard error of the mean (SEM) (*n* = 2).

Based on the observed biofilm characteristics (number of cells, PNAG and protein content) the HA- and TI-associated biofilms were further tested with different antibiotics. To this end, 18 and 66 h biofilms were exposed to four different antimicrobial (vancomycin, penicillin G, doxycycline, levofloxacin) agents for 2 or 24 h. As shown in Figure 7 the viable cell counts (CFUs) decreased in a statistically significant manner (*p* < 0.05 and *p* < 0.001) in all cases with the 24-h-long exposure when compared to the TSB-treated control coupons containing biofilm cells. A comparison of the used materials indicated that the 66-h-old biofilms on HA exposed to antibiotics for 2 h were more susceptible than biofilms on TI in all of the cases (Table 3). In general, biofilms formed on HA seemed to be more susceptible than in TI to antibiotics in most of the cases (in eight out of 11). When taking the biofilm age-related differences into account, the 66-h-old biofilms were found more susceptible than the 18-h-old biofilms in three out of 10 cases. In most of the cases (in seven out of 10), the younger biofilms (18 h) were more susceptible than the older (66 h) biofilms. Comparing the antibiotic treatment times indicated that increasing the exposure time from 2 to 24 h resulted in reduced chemotolerance in each biofilm age- and material-combination. From the tested antibiotics, levofloxacin at a final concentration of 90 µM was found as the most efficient in reducing the viable colonies from biofilms formed on both materials. The most efficient antibiotic treatment was obtained with 18-h-old biofilms exposed to levofloxacin for 24 h.

**Figure 8:**
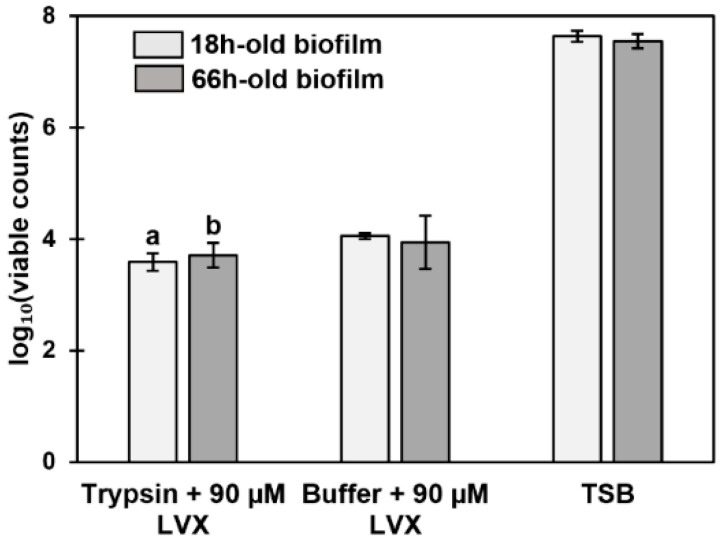
Viability of 18- and 66-h-old *S. aureus* ATCC 25923 biofilms treated with trypsin and 90 µM levofloxacin (LVX), triethylammonium bicarbonate buffer (TEAB) (trypsin buffer) and 90 µM levofloxacin or tryptic soy broth (TSB). The number of viable cells is expressed as log_10_ values of CFU (mL·cm^2^)^−1^. The statistical analysis was performed using unpaired *t*-tests with Welch’s correction. Highly significant change in viability (*p* < 0.001) compared to (a) TSB at 18 h time point and (b) TSB at 66 h time point. Error bars denote the standard error of the mean (SEM) (*n* = 2).

A combination treatment of trypsin and levofloxacin on 18- and 66-h-old biofilms formed on HA was tested. For this purpose, biofilms were first treated with trypsin (51.9 ng·µL^−1^) followed by a 90 µM levofloxacin treatment. Comparison of the results with the two controls (biofilms treated with TSB or 100 mM TEAB followed by 90 µM levofloxacin) revealed that the trypsin treatment alone did not benefit biofilm eradication (Figure 8). Statistically highly significant differences (*p* < 0.001) were only acquired when biofilms were treated with trypsin and levofloxacin in comparison with the TSB-treated biofilms. Additionally, there was no difference between the trypsin- and levofloxacin-treated 18- and 66-h-old biofilms, indicating that the protein-dependent matrix network was already well-established at the 18 h time point.

## 4. Discussion

It is known that biofilm formation of *S. aureus* depends, among other factors, on the functional characteristics of the indwelling medical device and the specific surface components of the bacterium. However, many biofilm studies have traditionally utilized PS-based surface materials as the substrate for promoting adherent/biofilm growth. The biggest drawback of such studies is that the obtained results are not directly applicable to other clinically relevant substrate materials. In addition, instead of systematic studies focusing on the biofilm substrates or the biofilm matrix components, the majority of the studies have investigated the role of the individual materials or specific surface-anchored components of the adhering *S. aureus*. The present study aimed at filling this gap in knowledge by exploring structural features of five clinically pertinent materials and complementing the findings with in-depth surfaceomics of *S. aureus* ATCC 25923 biofilms growing on those materials.

### 4.1. Structural Features and the Impact of PNAG on Biofilm Growth

The surface analyses of the tested substrate materials revealed considerable differences in roughness, which, however, did not correlate with the biofilm formation efficiency. This suggests that other physicochemical factors (such as surface charge and surface energy) might have played a role in the biofilm–substrate interactions and could explain the material-dependent changes in the number of exopolysaccharides (PNAG fraction) and protein. poly-*N*-acetyl-β-(1–6)-glucosamine (PNAG; also referred to as polysaccharide intercellular adhesin (PIA)) is a major exopolysaccharide in the *Staphylococcus aureus* biofilm matrix. It is partially deacetylated, and its synthesis is mediated by the *icaADBC* locus [64,65]. Biofilms on PS and HA materials had the highest amount of PNAG at the 18 h time point, but this content was reducing over time in all materials, with the deepest drop in TI biofilms. In contrast, the protein amount was increasing towards the end of the growth. The most significant increase in protein amount was detected with G and HA biofilms, while the highest elevation in the number of proteins was detected with TI and HA biofilms. These findings suggest that PNAG has a more important role than proteins in coordinating the adherence of the cells in young biofilms (18 h) on PS and HA. In contrast, the protein role would switch to be more crucial at the later stages of biofilm formation (contributing to material-specific adherence and/or maintaining biofilm integrity/stability). PNAG has a net positive charge, which, besides promoting intercellular interactions by binding to the negatively charged surfaces of neighboring cells, might additionally have stimulated the adherence to negatively charged surfaces (such as those provided by the hydrophilic PS). Shifts in extracellular pH due to metabolic fluctuations may have also affected the PNAG content, as pH has been shown to control the EPS stability and thereby the mechanical properties of the *S. aureus* SH1000 biofilm [66]. PNAG is known to affect the attachment of staphylococcal biofilms, pathogenesis [67], resistance to phagocytosis, polymorphonuclear leucocytes [68,69] and antibiotic tolerance [70]. We suggest that among the materials studied here, PS and HA provide the best support for PNAG-stimulated biofilm growth.

### 4.2. The Accessory and Core Surfaceomes of the S. aureus ATCC 25923 Biofilms

Greatest variations in surfaceomes were detected for TI-, HA- and G-associated biofilms already at the 18 h time point. Virulence factors such as hemolysin HlgB and EsxA (a chaperone and/or an adaptor protein, which interacts with host receptor proteins and interferes with host cell apoptotic pathways) [71,72], were found moderately abundant in all biofilms at the 42 and 66 h time points. Staphopain A (SspP), detected here in all biofilms (except on HA, at 18 h), has been proposed to increase bacterial persistence through, e.g., degrading the antimicrobial human peptide LL-37 [73]. Immunodominant antigens IsaA and IsaB were dominating proteins in all biofilm samples. IsaA has been proposed to have autolytic activity [74], while elevated IsaB-levels promote the virulence and persistence of MRSA [75]. Increased abundance of IsaA has also been reported for *Staphylococcus epidermidis* biofilms formed on TI [76], implying that this antigen could have mediated *S. aureus* biofilm formation on the selected materials in our study. Bifunctional autolysin (AtlA), identified on all materials at every time point, has been reported to be responsible for initial attachment in biofilm formation, bacterial cell wall degradation and cell separation during cell division [46,77]. In addition, this autolysin is also reported with a potential role in FnBP-mediated biofilm maturation [78]. Our findings suggest that FnBPA could be important in earlier stages of biofilm formation (in young biofilms, at the 18 h time point), whereas the abundance of FbnBP in older biofilms implies this adhesin might have a more prominent role in biofilm maturation.

Staphylococcal secretory antigen (SsaA) was one of the most abundant secreted antigens detected in all biofilm samples (except on TI); this immunodominant antigen is suggested to be involved in biofilm growth and biofilm-related infections [79,80]. The immunoglobulin-binding protein A (Spa), identified on all materials at every time point, has been shown to be in a pivotal role in biofilm formation [81]. Other potential surface adhesins, such as Ser-Asp repeat-containing protein C (SdrC), ClfA, ClfB, Bbp and EbpS detected here in initial stages of biofilm growth on one or several of the tested materials, could also play a role in promoting initial adherence to the tested materials. Glutamine synthetase, with an ability to bind fibronectin, laminin, collagen I and plasminogen [82], was specifically identified only in TI-associated biofilms, implying that biofilms formed on this substrate could interfere with the host immune defense system.

### 4.3. The Surface-Associated Moonlighters Dominate in All Studied Biofilms

The cytoplasmic proteins with predicted multitasking functions [82,83] formed the biggest group among all identified proteins, independent of the biofilm substrate used. The presence of cytoplasmic proteins in the extracellular milieu has been widely explained with cell lysis/leakage. However, this interpretation seems to be too simple, since several lines of evidence imply the existence of a yet unknown mechanism controlling cytoplasmic protein excretion. For example, it has been noted that cytoplasmic protein excretion is increased when the autolysins are up-regulated and peptidoglycan cross-linking is decreased [84]. Cell lysis in *S. aureus* biofilms has been shown to depend on the presence of the major autolysin Atl, the holin/antiholin system CidABC and LrgAB [78,85,86,87]. Atl has been shown to be strongly upregulated in moderately aged *S. aureus* biofilms, which resulted in strong lysis and accumulation of intracellular proteins in the biofilm matrix [47].

Thus, besides the conventional autolysin activity, AtlA can also control the excretion of cytoplasmic proteins (e.g., EfTU) to the cell surface; a process that is not random, but suggested to involve selection based on certain sequence motifs (e.g., α-helices) [84,87,88,89]. The Atl-mediated protein export could also occur in vivo in *S. aureus*, indicated by recent results in the post-arthroplasty joint infection model (hypodermic stainless-steel rod) where higher levels of cytoplasmic proteins were found, compared to the classical surface adhesins or other classically secreted proteins [48]. From the cytoplasmic proteins, GaPDH has been detected with increased abundances in *S. epidermidis* biofilms formed on TI [76], indicating that this moonlighter could be important for biofilm formation or stability of the formed biofilms. Besides their conventional roles in cytoplasm, the cytoplasmic proteins are reported to have also adhesive (to plasminogen, laminin, Caco-2-cell, mucin, EPS-derived mannan or rhamnose), immunomodulatory and/or biofilm formation stimulating functions [90,91,92,93,94].

Here, the r-protein moonlighters, bearing a high net positive charge with high affinity towards anionic cell wall components (eDNA and anionic metabolites), formed the biggest group of the identified moonlighting proteins. This is supported by [47], showing that r-proteins and several secreted virulence proteins (both having a strong positive charge) are embedded in the acidic *S. aureus* biofilm matrix. Acidic conditions are generated by the release of fermentation end-products as a response to oxygen limitation in the biofilms. In that study, the r-proteins were suggested to contribute to the pH-dependent stability of the biofilms. The r-proteins are classically involved in translation, but after associating with cell surfaces, they could also function as a defensive mechanism in response to external challenges originating from the host immune system, antibiotics or other challenges, as previously reported by [95]. This has been supported by a recent proteomic study reporting that the production of r-proteins is increased in response to an antibacterial agent, quinolonyl-oxazolidinone [96].

Our study also indicated the presence of several regulatory proteins normally acting in intracellular milieu by coordinating biofilm growth, virulence and/or drug resistance [97] at biofilm cell surfaces. *S. aureus* is also reported to use membrane vesicles (MVs) to transfer regulators, virulence factors and drug resistance enzymes in a protected and concentrated manner [98,99,100,101,102]. Regulator proteins SarR, SarS and Rot, as well as r-proteins, malate:quinone oxidoreductase 2 (MQO2), hemolysins, leukocidins, certain moonlighters (EfG, EfTU, chaperone protein DnaJ, Usp, PYK, ENO, PDHB, ATP synthase subunit beta), foldase protein A (PrsA) and penicillin-binding protein were previously identified from MVs isolated from *S. aureus* 06ST1048 [101]. Notably, leukocidins have been shown to be efficiently produced also during chronic infection in vivo [48], which further suggests that *S. aureus* can actively modulate the host immune system even protected within the biofilm. Thus, it can be hypothesized that these virulence factors are sorted into MVs for protected export, together with necessary moonlighters, aiming at maintaining the cohesion and viability of the biofilm community in vivo.

### 4.4. Older Biofilms Are Not Always More Tolerant Than Younger Biofilms

Our findings suggested that PS, G and PG could provoke protein-dependent antibiotic resistance, as the enzymes lysostaphin resistance protein A (LyrA) and methicillin resistance-associated FemA/B and FmtA [103,104,105] were detected in PS, PG and/or G biofilms already at the 18 h time point, implying that biofilms on other materials may be more susceptible to certain antibiotics. In our study, chemotolerance tests were executed with different antibiotics covering a broad spectrum of mechanisms of action: vancomycin (glycopeptide: inhibits cell wall synthesis by forming complexes with peptidoglycan precursors [106]), penicillin G (B-lactam: inhibits cell wall synthesis via preventing peptidoglycan polymerization [107]), doxycycline (tetracycline: inhibits protein synthesis by binding to the 30S subunit of the bacterial ribosome [108]) and levofloxacin (fluoroquinolone: inhibits bacterial DNA gyrase and topoisomerase IV in Gram-negative and Gram-positive bacteria, respectively, and blocking DNA replication [109]). Furthermore, levofloxacin, vancomycin and doxycycline are used as a part of treatment regimen in managing prosthetic joint infections caused by *Staphylococcus* spp. Usually, vancomycin is administered intravenously for the first two weeks after the surgical therapy (if oxacillin-, methicillin- or rifampicin-resistance has been observed), while doxycycline and levofloxacin are given per oral as a continuation therapy (a total duration of antibiotic treatment is 12 weeks) [110]. Our results were in accordance with the results obtained by [111], where several antibiotics were tested against *Staphylococcus aureus* ATCC 25923. Therein, levofloxacin was the most effective, followed by doxycycline, penicillin G and vancomycin (least active). In our study, levofloxacin was the most effective, while the second most active was penicillin G or doxycycline depending on the biofilm age, used substrate material or exposure time of the antibiotic. Least active was vancomycin, as well. Furthermore, in another study, doxycycline displayed higher activity than vancomycin against different *Staphylococcus aureus* clinical isolates [112].

The results also revealed that in many cases, biofilms formed on HA were more susceptible to antibiotics than biofilms on TI. Unexpectedly, the 66 h biofilms were not always more tolerant than those grown for 18 h, which may suggest that other surface factors, such as the r-protein moonlighters or other moonlighting proteins could have contributed to the observed phenotypes.

### 4.5. Several Biofilm Surfaceome Proteins Are Important for Successful Infection

Hemolysins, leukocidins, stress proteins (AhpC/F, Usp, SodA), resistance (FmtA), chaperones (ClpL, ClpC, ClpB, DnaK and GroEL) and response regulators (VraR, CodY and CcpA) identified here already at 18-h-old biofilms were recently suggested to be involved in host–pathogen interactions in vivo [113]. Several of these proteins were also identified as secreted and/or matrix-associated proteins in an implant-associated biofilm in vivo infection model [48]. In addition, the Clp family proteins are reported to be important for biofilm formation and virulence [114]. The appearance of ClpP at the cell surface is interesting, as the enzyme can be activated by an acyldepsipeptide antibiotic into a non-specific protease capable of killing *S. aureus* persisters [115].

## 5. Conclusions

The present study indicated the importance of PNAG and dedicated cell wall/membrane-anchored proteins during the initial stages of biofilm formation. The recycling of cytoplasmic proteins as moonlighting components could benefit biofilm population by increasing the integrity, stability and drug resistance of the cells. As the development of the protein matrix was slowest on HA and TI, our study proposes that these substrates could provide a good starting point for generating new clinical materials with enhanced anti-biofilm features. Prior to this, as biofilms formed on HA were found to reach sufficient maturity already at the 18 h time point, in-depth surfaceome analysis on this material should be investigated also at earlier time points. Classical surface proteins have been considered as the most attractive targets in drug design against bacterial pathogens. However, this research should be expanded to also include non-classical moonlighters, as many of these can contribute to virulence and drug tolerance. Thus, understanding mechanisms coordinating the moonlighting activity would not only provide fundamental insights into bacterial gene regulation, but it may also shed better insight into new strategies aimed at designing novel anti-biofilm agents/materials.

## Figures and Tables

**Table 1:** Selected roughness parameters of materials used in the assays. S_dr_ describes the relative increase in surface area compared to the flat surface. V_v_ reflects void volume, describing the openness of the surface or surface porosity.

Material	S_dr_ (%)	V_v_ (µm^3^/µm^2^)
Borosilicate glass	0.3 ± 0.1	0.0048 ± 0.001
Plexiglass	123 ± 20	0.62 ± 0.07
Hydroxyapatite	58 ± 10	0.75 ± 0.08
Titanium	9.0 ± 1.1	0.19 ± 0.02
Polystyrene	3.0 ± 0.4	0.012 ± 0.002

**Table 2:** A heatmap comparing the intensity values (log_10_) of selected protein identification.

Protein Name	Acc. No. ^a^	18 h					42 h					66 h				
		PS	G	HA	PG	TI	PS	G	HA	PG	TI	PS	G	HA	PG	TI
Bifunctional autolysin—AtlA	Q6GI31															
Immunoglobulin G-binding protein A—Spa	P38507															
Immunoglobulin-binding protein—Sbi	Q6GE15															
Immunodominant antigen B—IsaB	Q6GDG4															
Immunodominant antigen A—IsaA	Q6GDN1															
Malate:quinone oxidoreductase 2—MQO2	Q6GDJ6															
Leukocidin-like protein 2—Luk2	Q6GF49															
Leukocidin-like protein 1—Luk1	Q6GF50															
Non-heme ferritin—FtnA	Q99SZ3															
Foldase protein A—PrsA	Q6GFL5															
Thioredoxin	Q6GHU0															
Staphylococcal secretory antigen—SsaA	Q99RX4															
Putative dipeptidase SAR1836	Q6GFV0															
Clp protease proteolytic subunit—ClpP	Q6GIM3															
Clp ATPase ClpC	Q99W78															
Clp ATPase ClpL	Q6GDQ0															
Clp ATPase ClpB	Q6GIB2															
Gamma-hemolysin component B—HlgB	Q6GE12															
Protein RecA	Q6GHF0															
Bone sialoprotein-binding protein—Bbp	Q6GJA6															
Clumping factor B—ClfB	Q6GDH2															
Clumping factor A—ClfA	Q6GIK4															
Delta-hemolysin—HglD	Q6GF37															
Virulence factor—EsxA	Q99WU4															
ATP-dependent protease ATPase—HslU	Q6GHI1															
Elastin-binding protein—EbpS	Q6GGT1															
Fibrinogen-binding protein—FbnBP	Q6GHS9															
Fibronectin-binding protein A—FnBPA	Q6GDU5															
Ser-Asp repeat-containing protein C—SdrC	Q6GJA7															
Ser-Asp repeat-containing protein D—SdrD	Q8NXX6															
Catabolite control protein A—CcpA	Q6GFX2															
Response regulator—CodY	Q6GHI0															
Response regulator—SarA	Q7A732															
Response regulator—Rot	Q9RFJ6															
Response regulator—SarR	Q9F0R1															
Response regulator—SarS	Q7A872															
Response regulator—VraR	Q7A4R9															
Response regulator—SaeR	Q99VR7															
Response regulator—MsrR	Q99Q02															
Response regulator—MraZ	Q6GHQ7															
Response regulator—LytR	P52078															
Response regulator—NrdR	Q6GG20															
Response regulator—GraR	Q6GJ11															
HTH-type transcriptional regulator—MgrA	Q99VT5															
Redox-sensing repressor—Rex	Q6GF26															
SOS response repressor—LexA	Q9L4P1															
Oxygen regulatory protein—NreC	Q99RN8															
Regulatory protein—Spx	Q6GI88															
Histidine protein kinase—SaeS	Q99VR8															
RNA polymerase sigma factor SigA	Q99TT5															
Anti-sigma-B factor antagonist—RsbV	Q6GF07															
Iron-regulated surface determinant—IsdB	Q6GHV7															
Lysostaphin resistance protein A—LyrA	Q6GEA0															
Methicillin-resistance protein—FmtA	Q6GI27															
Conserved virulence factor B—CvfB	Q99U93															
DegV domain-containing protein SAR1438	Q6GGY2															
Signal transduction protein TRAP	Q6GFM2															
Staphopain A (cysteine protease)—SspP	Q6GFE8															
Ferrochelatase—HemH	Q6G8A3															
Phospholipase C—PlC	Q5HEI1															
Methicillin resistance-associated—FemA	Q99UA7															
Methicillin resistance-associated—FemB	Q6GH30															
Probable cell wall amidase—LytH	Q7A588															
ATP-dependent protease subunit—HslV	Q6GHI2															
CtpA-like serine protease	Q6GGY8															
HtrA-like serine protease	Q6GI62															
Hydrolase encoded by the agr operon	P55177															
Probable thiol peroxidase	Q6GFZ4															
Uncharacterized oxidoreductase SAR2567	Q6GDV6															
Peptide methionine sulfoxide reductase MsrB	Q6GGY4															
Heme-dependent peroxidase (SAV0587)	Q99W24															
Thioredoxin reductase	Q6GB66															
NADPH-dependent oxidoreductase	Q6GJR6															
Multicopper oxidase—Mco	Q6GIX3															
Nitric oxide synthase oxygenase	Q6GFE2															
Putative NAD(P)H nitroreductase (SAV2523)	Q99RB2															
FMN-dependent NADPH-azoreductase	Q99W49															
Staphylocoagulase—Coa	P17855															
Iron-sulfur cluster repair protein—ScdA	Q6GK53															
Urease accessory protein G—UreG	Q99RX9															
ATP synthase epsilon chain	Q6GEX3															
ATP synthase subunit delta	Q6GEW9															
ATP synthase gamma chain	Q99SF4															
30S ribosomal protein S1	Q6GGT5															
30S ribosomal protein S10	Q931G5															
30S ribosomal protein S11	Q6GEK8															
30S ribosomal protein S12	Q6GJC3															
30S ribosomal protein S13	Q6GEK7															
30S ribosomal protein S15	Q99UJ9															
30S ribosomal protein S16	Q6GHJ7															
30S ribosomal protein S17	Q8NVB4															
30S ribosomal protein S18	Q6GJV1															
30S ribosomal protein S19	Q6GEI7															
30S ribosomal protein S2	Q6GHH9															
30S ribosomal protein S20	Q99TR3															
30S ribosomal protein S21	Q6GGC5															
30S ribosomal protein S3	Q6GEI9															
30S ribosomal protein S4	Q6GFY8															
30S ribosomal protein S5	Q6GEK0															
30S ribosomal protein S6	Q6GJV3															
30S ribosomal protein S7	Q6GJC2															
30S ribosomal protein S8	Q6GEJ7															
30S ribosomal protein S9	Q6GEL8															
50S ribosomal protein L1	Q6GJD0															
50S ribosomal protein L10	Q6GJC9															
50S ribosomal protein L11	Q6GJD1															
50S ribosomal protein L13	Q99S51															
50S ribosomal protein L14	Q99S31															
50S ribosomal protein L15	Q6GEK2															
50S ribosomal protein L16	Q99S28															
50S ribosomal protein L17	Q99S46															
50S ribosomal protein L18	Q99S37															
50S ribosomal protein L2	Q6GEI6															
50S ribosomal protein L20	Q6GG27															
50S ribosomal protein L21	Q99TK6															
50S ribosomal protein L22	Q99S26															
50S ribosomal protein L23	Q99S23															
50S ribosomal protein L24	Q6GEJ4															
50S ribosomal protein L25	Q99WA2															
50S ribosomal protein L27	Q931Q3															
50S ribosomal protein L28	Q6GHL1															
50S ribosomal protein L29	Q6GEJ1															
50S ribosomal protein L3	Q6GEI3															
50S ribosomal protein L30	Q6GEK1															
50S ribosomal protein L31	Q6GEV5															
50S ribosomal protein L35	Q6GG26															
50S ribosomal protein L4	Q6GEI4															
50S ribosomal protein L5	Q99S33															
50S ribosomal protein L6	Q99S36															
50S ribosomal protein L7/L12	Q6GJC8															
50S ribosomal protein L9	Q6GKT0															
Elongation factor Tu—EfTU	Q6GJC0															
Elongation factor G—EfG	Q6GJC1															
Translation initiation factor IF-3—InfC	Q6GG25															
Translation initiation factor IF-2—InfB	Q6GHG6															
Elongation factor P—EfP	Q6GGH0															
Glyceraldehyde-3-phosphate dehydrogenase	Q6GIL8															
Enolase—ENO	Q6GIL4															
Phosphoglycerate kinase—PGK	Q6GIL7															
Pyruvate kinase—PYK	Q6GG09															
Fructose-bisphosphate aldolase class 1—FBA	Q6GDJ7															
Pyruvate dehydrogenase E1—PDHB	Q6GHZ1															
Triosephosphate isomerase—TPI	Q6GIL6															
ATP-dependent 6-phosphofructokinase—PFK	Q6GG08															
2,3-phosphoglycerate mutase—PPGM	Q6GE17															
Aconitase A—AcnA	Q6GH55															
L-lactate dehydrogenase 1—L-LDH	Q6GK73															
D-lactate dehydrogenase—D-LDH	Q6GDS2															
Alkaline shock protein 23—Asp23	Q6GEP7															
Alcohol dehydrogenase—ADH	Q99W07															
Trigger factor—Tf	Q6GG30															
DNA-directed RNA polymerase—RpoB	Q6GJC5															
Alkyl hydroperoxide reductase—AhpC	Q6GJR7															
Alkyl hydroperoxide reductase—AhpF	Q6GJR8															
Chaperone protein—GroEL	Q6GF43															
Chaperone protein—DnaK	Q6GGC0															
Chaperone protein—DnaJ	Q6GGC1															
10 kDa chaperonin	Q6GF42															
Universal stress protein (SAV1710)—Usp	Q99TF3															
Superoxide dismutase [Mn/Fe] 1—SodA	Q6GGE6															
DNA mismatch repair protein—MutL	Q93T05															
Thermonuclease	Q5HHM4															
Glutamine synthetase	Q6GHC6															

Color gradient from red to green is used to indicate decreasing intensity values. 
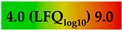
 ^a^ Acc. No., accession numbers were retrieved from the UniProt protein database. In red are proteins involved in adherence and/or adherent growth (biofilm formation), and in blue are adhesive moonlighters.

**Table 3:** The chemotolerance of *S. aureus* ATCC 25923 as quantified using the log reduction of viable counts. The 18-h- and 66-h-old biofilms were formed on hydroxyapatite (HA) and titanium (TI), and exposed to penicillin G, levofloxacin, doxycycline and vancomycin.

Biofilm Age	Biofilm Formed on	Exposure Time	Penicillin G (2.0 µM) ^a^	Levofloxacin (90.0 µM) ^a^	Doxycycline (4.0 μM) ^a^	Vancomycin (5.0 µM) ^a^
**18 h**	HA	2 h	0.10 ± 0.28	0.56 ± 0.06	0.08 ± 0.15	0.03 ± 0.31
**18 h**	TI	2 h	0.01 ± 0.19	1.39 ± 0.05 ***, ⱡ ⱡ ⱡ	0.59 ± 0.21 *, ⱡ	−0.17 ± 0.25
**18 h**	HA	24 h	2.32 ± 0.18 ***, ⱡ ⱡ ⱡ, Ω	3.09 ± 0.04 ***, ⱡ ⱡ ⱡ, Ω Ω Ω	2.00 ± 0.14 *, ⱡ, Ω Ω Ω	0.65 ± 0.09 Ω
**18 h**	TI	24 h	1.28 ± 0.08 ⱡ	2.18 ± 0.15 ⱡ ⱡ ⱡ, Ω Ω Ω	1.08 ± 0.20 Ω	0.33 ± 0.09
**66 h**	HA	2 h	0.42 ± 0.04 *	0.64 ± 0.24 *, ⱡ	0.31 ± 0.08	0.61 ± 0.11 *, ⱡ
**66 h**	TI	2 h	0.07 ± 0.15	0.46 ± 0.08	0.39 ± 0.06	0.24 ± 0.12
**66 h**	HA	24 h	1.82 ± 0.24 *, Ω Ω Ω	2.35 ± 0.07 ***, Ω	1.32 ± 0.20 Ω	0.88 ± 0.38
**66 h**	TI	24 h	0.94 ± 0.18 Ω	1.76 ± 0.14 Ω Ω Ω	1.62 ± 0.04 *, ⱡ, Ω Ω Ω	1.67 ± 0.03 Ω

^a^ The numbers refer to logR values (±SEM) indicating the difference between antibiotic- and medium-treated coupons. *, *p* < 0.05 and ***, *p* < 0.001; differences between HA and TI, when the biofilm age and exposure time are the same. ⱡ, *p* < 0.05 and ⱡ ⱡ ⱡ, *p* < 0.001; differences between the 18- and 66-h-old biofilms when the exposure times and the materials are same. Ω, *p* < 0.05 and Ω Ω Ω, *p* < 0.001; differences between exposure times (2 h and 24 h) when the biofilm age and the materials are the same. The statistical analyses were performed using unpaired t-tests with Welch’s correction.

## Supplementary Materials

The following are available online at https://www.mdpi.com/2076-2607/7/12/584/s1. Figure S1: The viability of *S. aureus* ATCC 25923 biofilm cells treated with trypsin or 100 mM TEAB buffer (control). Figure S2: Length-scale dependent roughness of the materials. Figure S3: Venn diagrams indicating the number of proteins specific and common to the indicated materials at different time points of growth. Table S1: The MaxQuant output-file showing the identified proteins after searching the MS/MS raw data against the UniProt *Staphylococcus* protein database. Table S2: The identified matrix-associated surfaceomes from *S. aureus* ATCC 25923 biofilms formed on different substrates at indicated time points.
