# Supplementary figures and images for: Structural and Functional Dynamics of Staphylococcus aureus Biofilms and Biofilm Matrix Proteins on Different Clinical Materials
Source: Microorganisms. 2019 Nov 20;7(12):584. doi: 10.3390/microorganisms7120584 (PMC6955704; doi:10.3390/microorganisms7120584)

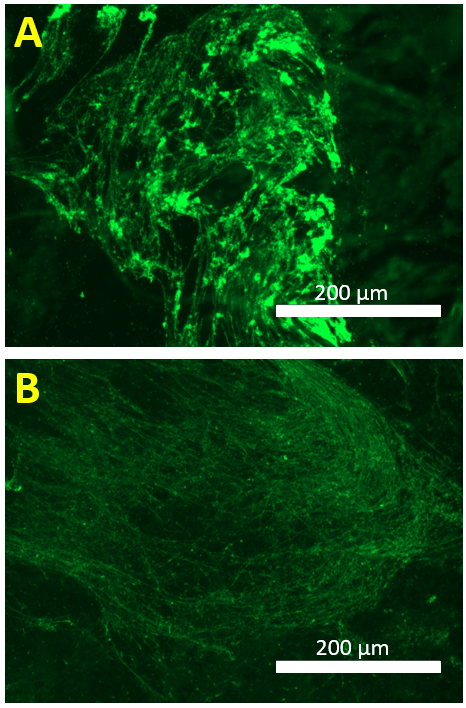

Supplement: Supplementary file 1 [file microorganisms-07-00584-s001.zip › FIG. 4_proof-read.png]

A

G

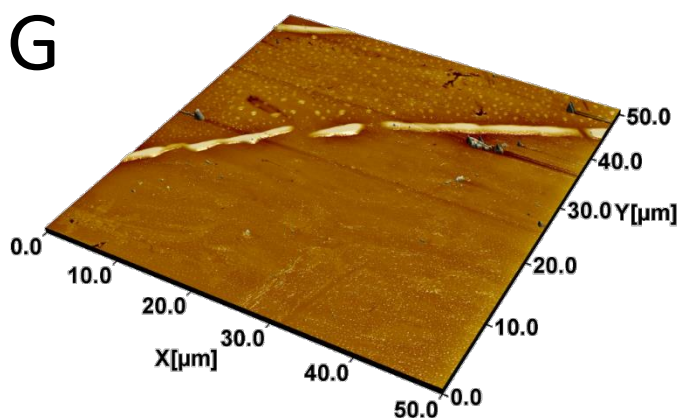

B

G

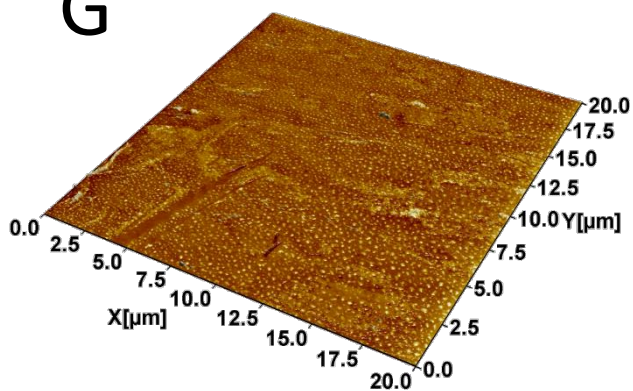

PG

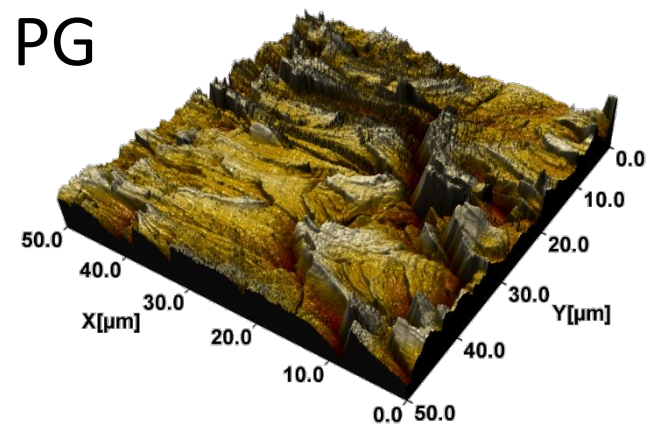

PG

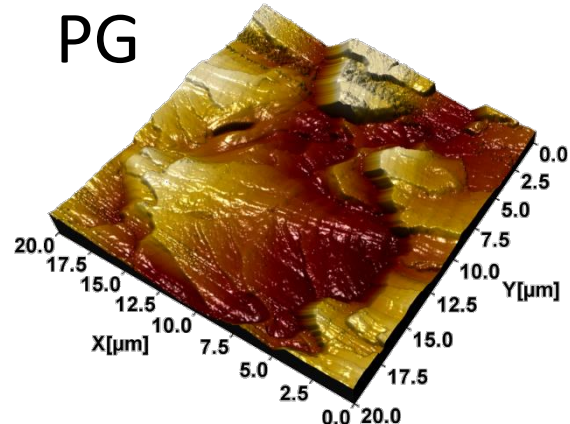

HA

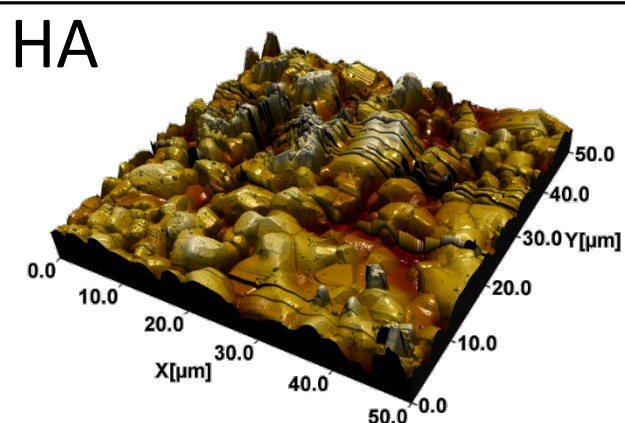

HA

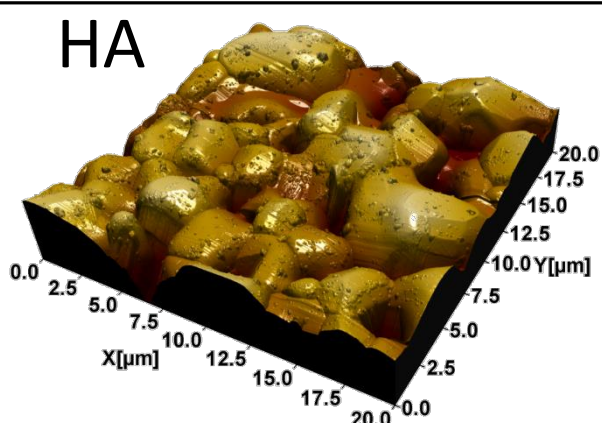

TI

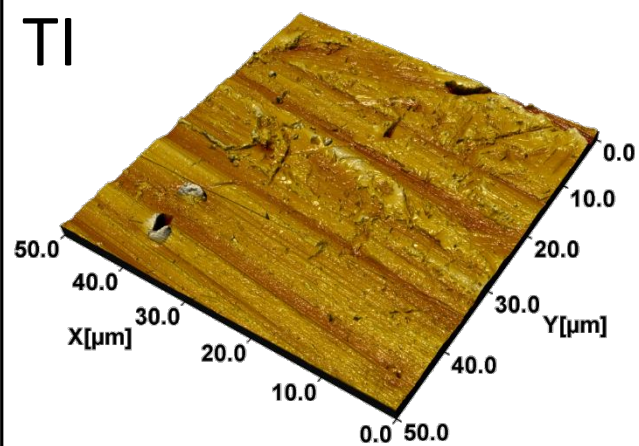

TI

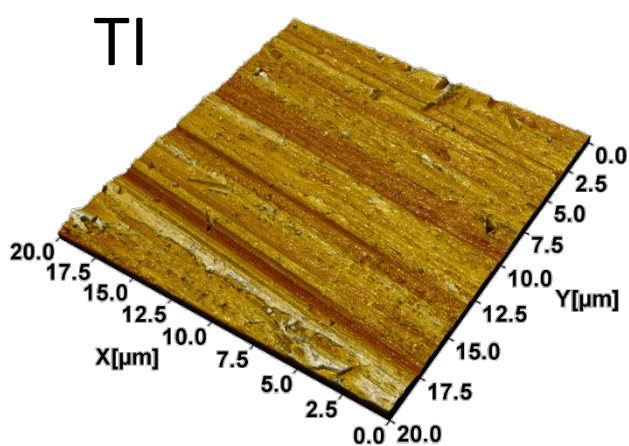

PS

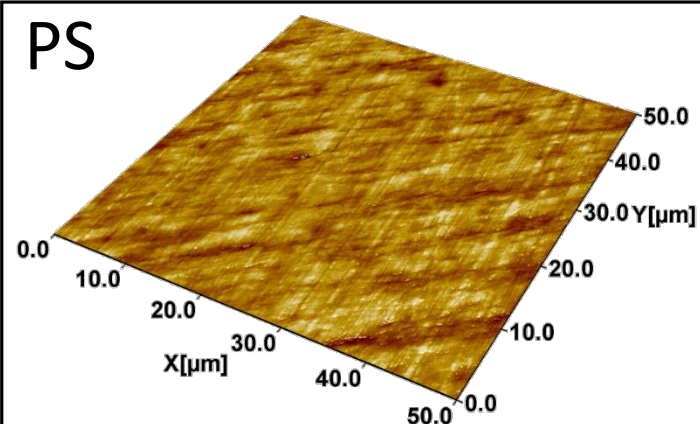

PS

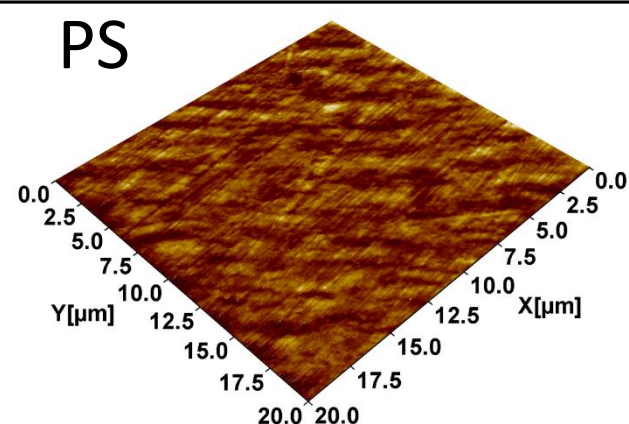

Supplement: Supplementary file 1 [file microorganisms-07-00584-s001.zip › Figure 1.pdf]

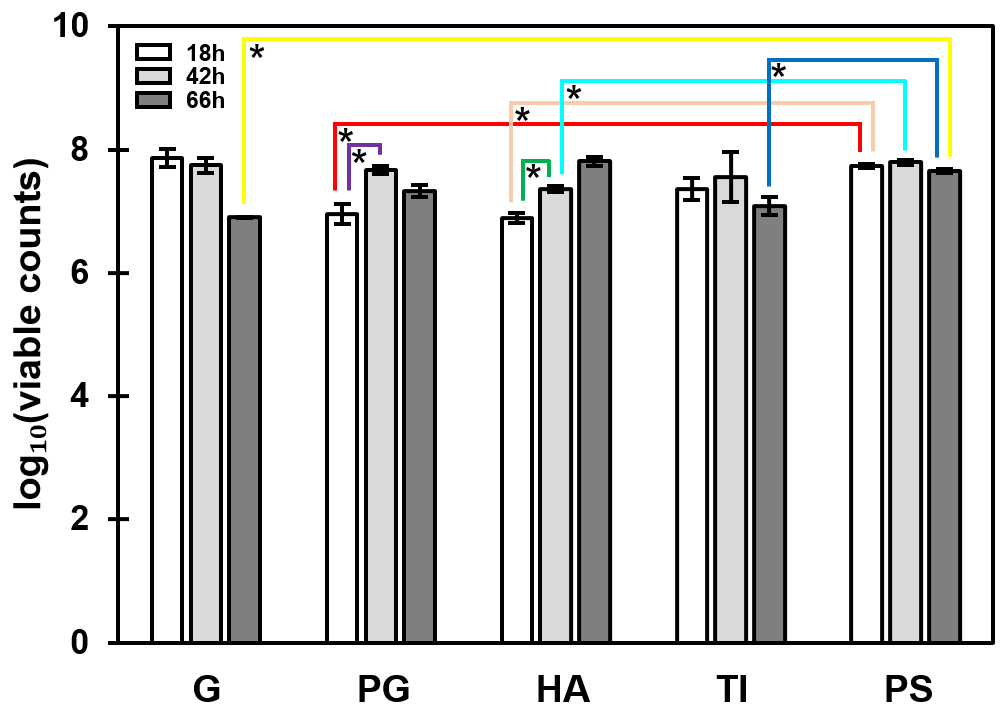

Supplement: Supplementary file 1 [file microorganisms-07-00584-s001.zip › Figure 2_corrected.png]

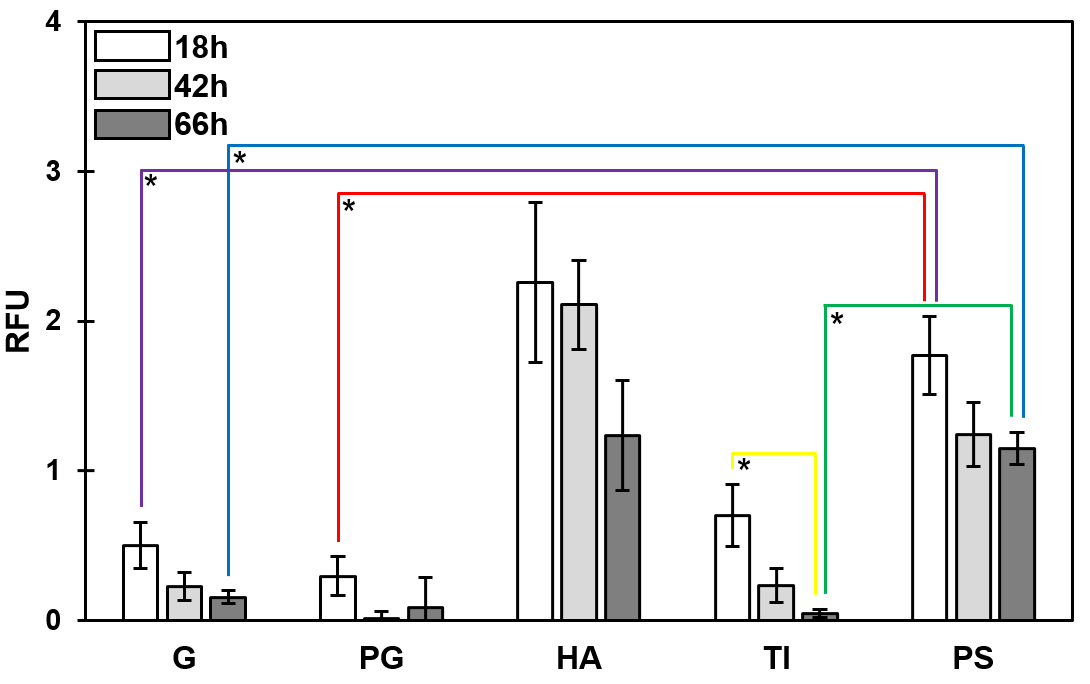

Supplement: Supplementary file 1 [file microorganisms-07-00584-s001.zip › Figure 3_corrected.png]

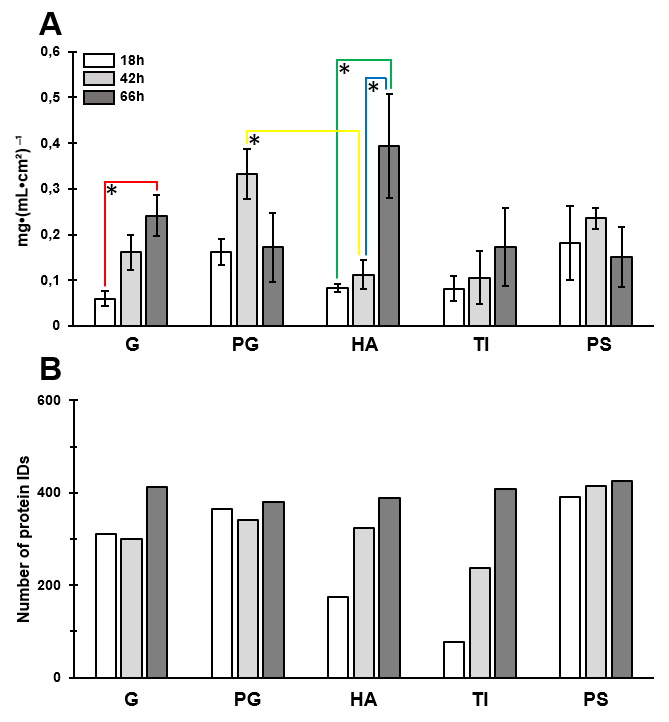

Supplement: Supplementary file 1 [file microorganisms-07-00584-s001.zip › Figure 5_corrected.png]

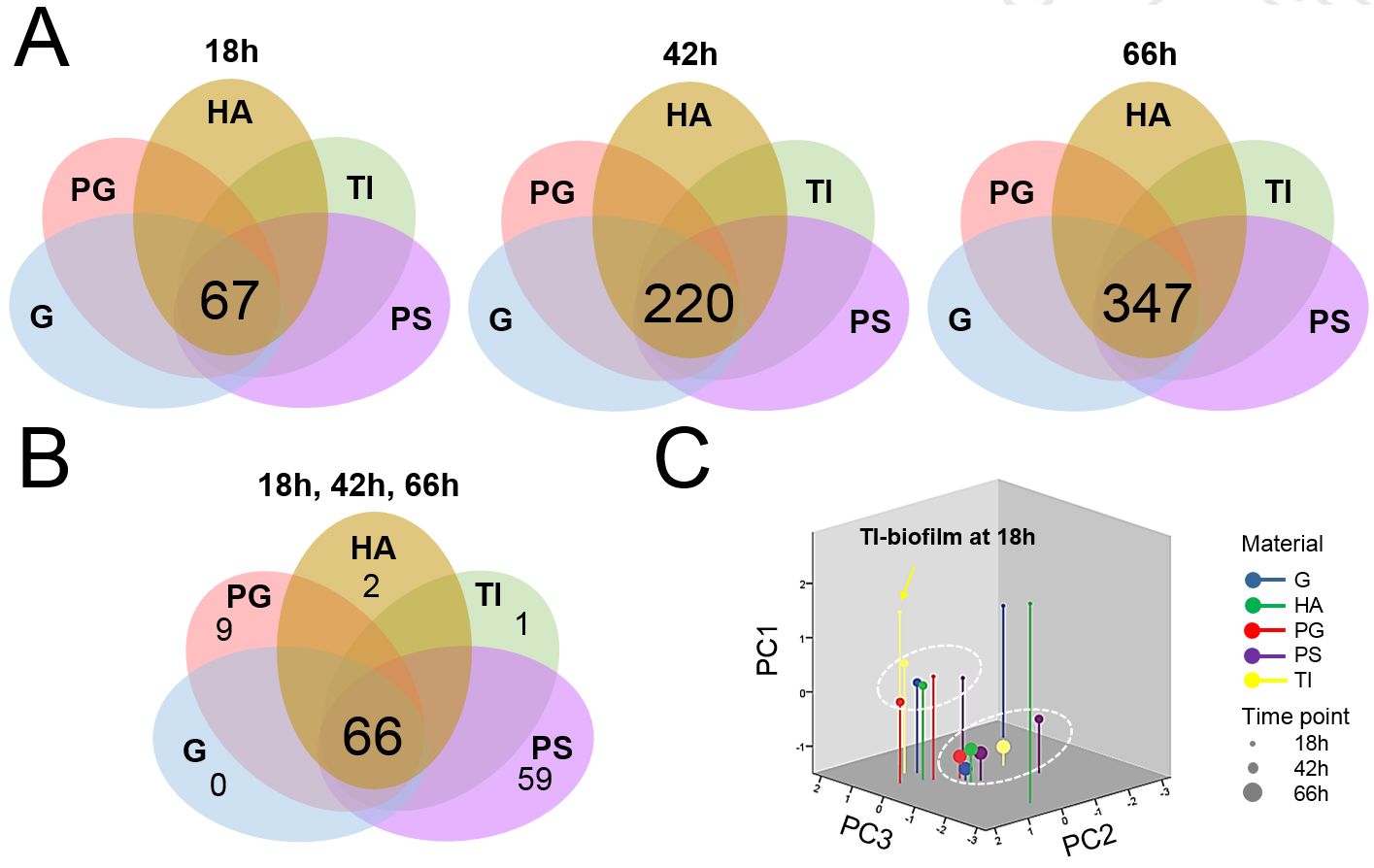

Supplement: Supplementary file 1 [file microorganisms-07-00584-s001.zip › Figure 6.png]

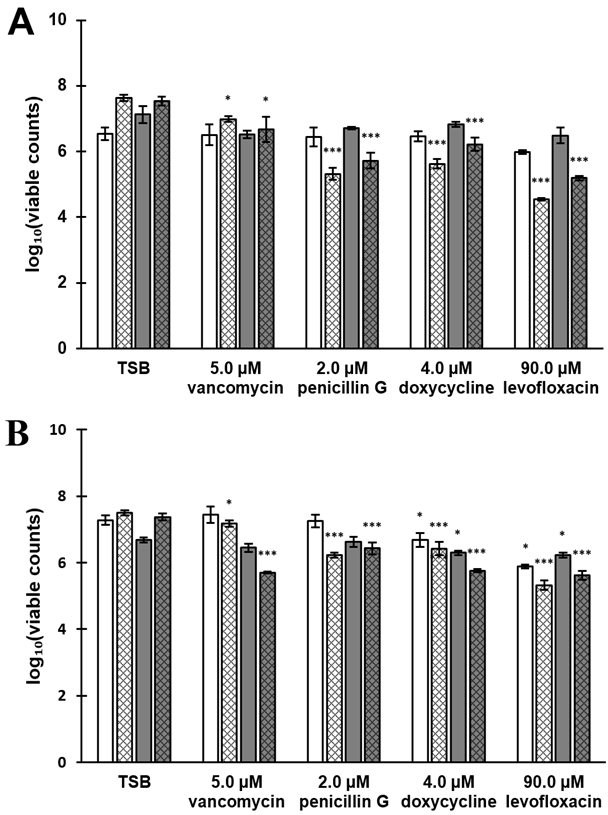

Supplement: Supplementary file 1 [file microorganisms-07-00584-s001.zip › Figure 7.png]

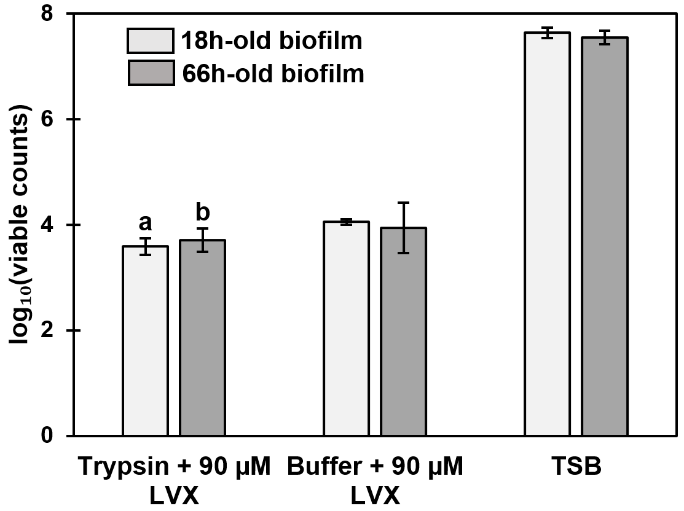

Supplement: Supplementary file 1 [file microorganisms-07-00584-s001.zip › Figure 8.png]
